# Analysis of Ultra Low Genome Conservation in *Clostridium difficile*

**DOI:** 10.1371/journal.pone.0015147

**Published:** 2010-12-08

**Authors:** Joy Scaria, Lalit Ponnala, Tavan Janvilisri, Weiwei Yan, Lukas A. Mueller, Yung-Fu Chang

**Affiliations:** 1 Department of Population Medicine and Diagnostic Sciences, College of Veterinary Medicine, Cornell University, Ithaca, New York, United States of America; 2 Center for Advanced Computing, Cornell University, Ithaca, New York, United States of America; 3 Department of Biology, Faculty of Science, Mahidol University, Bangkok, Thailand; 4 Boyce Thompson Institute for Plant Research, Ithaca, New York, United States of America; University of Liverpool, United Kingdom

## Abstract

Microarray-based comparative genome hybridisations (CGH) and genome sequencing of *Clostridium difficile* isolates have shown that the genomes of this species are highly variable. To further characterize their genome variation, we employed integration of data from CGH, genome sequencing and putative cellular pathways. Transcontinental strain comparison using CGH data confirmed the emergence of a human-specific hypervirulent cluster. However, there was no correlation between total toxin production and hypervirulent phenotype, indicating the possibility of involvement of additional factors towards hypervirulence. Calculation of *C. difficile* core and pan genome size using CGH and sequence data estimated that the core genome is composed of 947 to 1,033 genes and a pan genome comprised of 9,640 genes. The reconstruction, annotation and analysis of cellular pathways revealed highly conserved pathways despite large genome variation. However, few pathways such as tetrahydrofolate biosynthesis were found to be variable and could be contributing to adaptation towards virulence such as antibiotic resistance.

## Introduction

*Clostridium difficile* is a gram-positive spore-forming anaerobic bacterium with a wide host range [1]. In recent years it has emerged as a major nosocomial pathogen. The complications arising from its infection are collectively called *C. difficile-*associated disease (CDAD) [2], [3]. In its simplest form CDAD can lead to mild diarrhea, but in the extremes it results in serious sequelae, toxic megacolon, intestinal perforation, peritonitis or death [2]. Several CDAD outbreaks in the past decade in Europe and North America have been attributed to the emergence of hypervirulent *C. difficile* strains belonging to PCR ribotype 027/pulse-field type NAP1 (027/NAP1) which produce elevated levels of toxins A and B, the primary virulence factors of this bacterium [4]–[9].

The first sequenced *C. difficile* genome was of a strain isolated from a patient with pseudomembranous colitis in Zurich, Switzerland [10]. This genome contains a large number of mobile genetic elements and very low genome conservation when compared to other *C. difficile* strains and also to other members of *Clostridia*. A microarray-based comparative genome hybridization (CGH) of this strain against 8 other *C. difficile* strains showed that the up to 61% of the total coding sequences (CDS) were absent from at least one strain tested [10]. A subsequent CGH comparison of 75 *C. difficile* strains revealed that only 19.7% genes were shared by all strains studied [11]. In our recent CGH analysis of a similar number of strains showed that the core genome of this species could be as low as 16% [12]. The absent/divergent genes in the tested strains were distributed across the entire *C. difficile* genome and across all gene functional categories [12]. It is surprising that the “core gene set” containing conserved genes in all tested *C. difficile* strains is unusually low and to our knowledge it is probably the smallest core genome reported for any bacterial species so far. For example, in other bacterial species with a large amount of genome variation such as *Helicobacter pylori* and *Campylobacter jejuni*, the core genomes were reported to be 72.5% and 59.2% of their total genomes, respectively [13]. Recently, genome sequencing of additional *C. difficile* strains further confirmed the large-scale genome variation in this species [14], [15]. Considering the ultra low genome conservation in *C. difficile*, in this study, we conducted a detailed analysis of the genome variation by integrating microarray CGH data, comparative genome sequencing and genome pathway data. First we compared 167 *C. difficile* strains using CGH data and the results were then corroborated using comparative genome sequencing of 4 divergent strains from the CGH-analyzed strains and also by comparing these genomes with 11 other *C. difficile* genomes. Finally the impact of the genome variation on *C. difficile* pathways was analyzed by pathway reconstruction and annotation using high-throughput experimental data as well as overlaying CGH data onto the curated pathways.

## Results and Discussion

### Analysis of genome variation using CGH data

**Figure 1:**
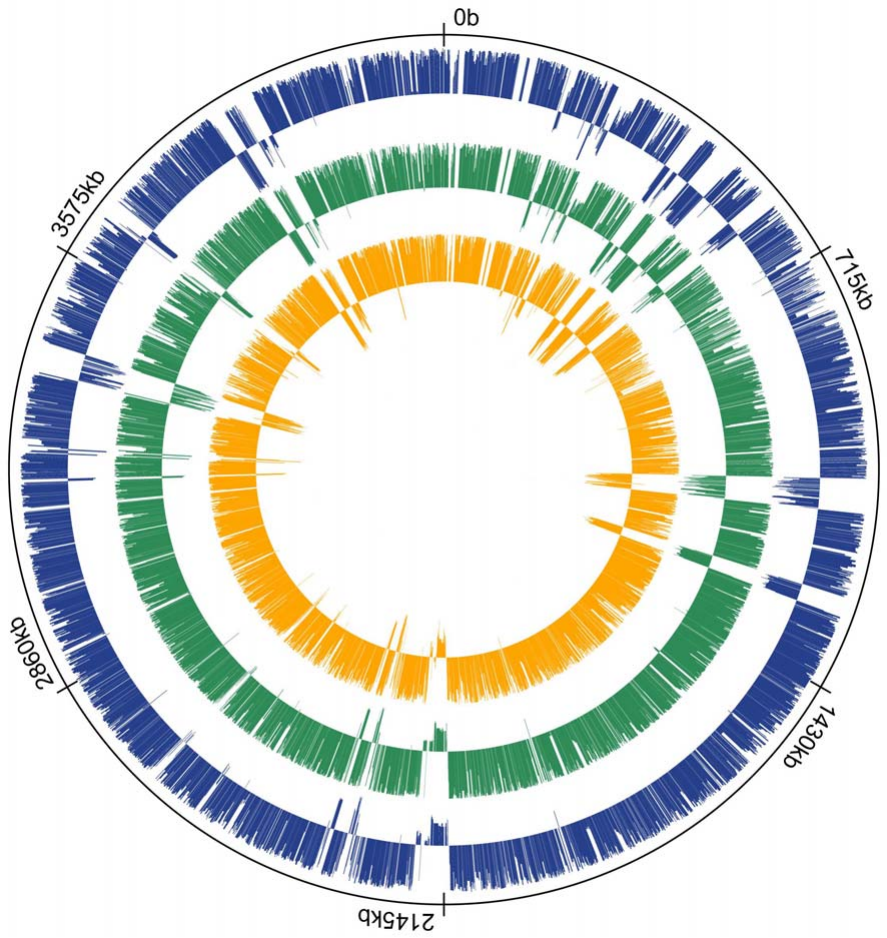
Comparison of CGH data from different microarray platforms and projection of CDS variability across *C. difficile* genome. From outside to inside: Ring 1 (Solid line). Molecular clock drawn using *C. difficile 630* genome. Ring 2 (Blue). LPI calculated using strains from Janvilisri et al. data. Ring 3 (Green). LPI calculated using Stabler *et al* data. Ring 4 (Orange). LPI calculated using combined dataset (Table S3).

The microarray dataset in this study comprised CGH comparison of 167 strains in total from three different sources. First, we carried out CGH analysis of 18 *C. difficile* isolates from human, bovine, canine and food origin (Table S1) using our *C. difficile* spotted arrays (Gene expression omnibus (GEO) platform: GPL6118). Although CGH analysis of isolates from human and bovine origins has been carried out previously, this study is the first CGH comparison of food and canine *C. difficile* isolates. The other sources of the data are meta-analyses of CGH data from Stabler *et al* study [11] and a former study by our group [12]. Since these two studies utilized PCR-based and 70-mer oligonucleotide spotted array platforms, we therefore evaluated the comparability of both datasets before combining them. Using GACK transformed CGH data (Table S2), we devised an index called Locus Plasticity Index (LPI). The LPI value for a CDS represents a numerical value of the degree of each gene's variation. Under this scheme, when a gene is present in all strains in the comparison, it would receive a value of +1.0 and when the gene is absent from all strains except the reference strain, the value will be −1.0. The range in between will be indication of degree of divergence. A projection of LPI values for all CDS in *C. difficile* 630 calculated separately and jointly from these two platforms is given in Figure 1. The results revealed that both platforms were highly comparable with a high correlation coefficient of 0.88 (Table S3). Consistent with the previous findings, the variation was distributed across the entire *C. difficile* genome. The regions with negative LPI values corresponded to those of mobile genetic elements. Deletions in several loci have been proposed to be specifically associated with hypervirulent strains [11]. However, a closer examination of our dataset showed that this is not always the case. For example, the loci of CD0719-CD0724 were intact in some of the 027/NAP1 strains in our collection. The genome of one of these strains, QCD-32g58, has been sequenced and these results also support the microarray data. Two studies of CGH of *C. difficile* yielded slightly different predictions of the core genome size [11], [12]. Here, the genes with high LPI values represent the core genes or genes with limited sequence variation.

Although there are a total of 3,971 CDS in *C. difficile* 630, we could only map 3,574 CDS in our dataset due to microarray platform differences. Of these, 151 CDS had an LPI value of +1.0. The genes with LPI values in the range of 0.95 to 0.99 received the lower values because 1–3 strains in either of the datasets classified these loci as divergent. This discrepancy arises due to the difference in microarray platform used. Stabler's data were based on PCR product arrays which although provided higher signals as full CDS were printed, but were prone to cross-hybridization. Microarrays used by our group were 70mer-based spotted array that produced more stringent results. However when the probes representing a CDS span a hyper-variable region of the gene, the signal could be reduced or lost and the CDS could be classified as being absent/divergent. This reflected in the core gene estimations in the previous analysis. According to Stabler *et al* the *C. difficile* core genome is 19.7% of the total 630 genome [11] while Janvilisri *et al* estimated it to be 16% of the total CDS [12]. Both estimates heavily lean towards eliminating false positives. For example, *slpA* gene encoding a major cell surface protein that forms a paracrystalline array in *C. difficile* contains a conserved region and a variable region [16]–[18]. This gene was classified as absent in both the previous analysis. Similarly it has been pointed out that due to several point mutations in *tcdB*, toxin B-positive strains were marked as negative as this is beyond the detection specificity of microarrays [11]. In the ternary classification scheme such genes are not included in the core gene set and the LPI index scheme would be a better representation of variability in such instances.

### Transcontinental strain comparison

**Figure 2:**
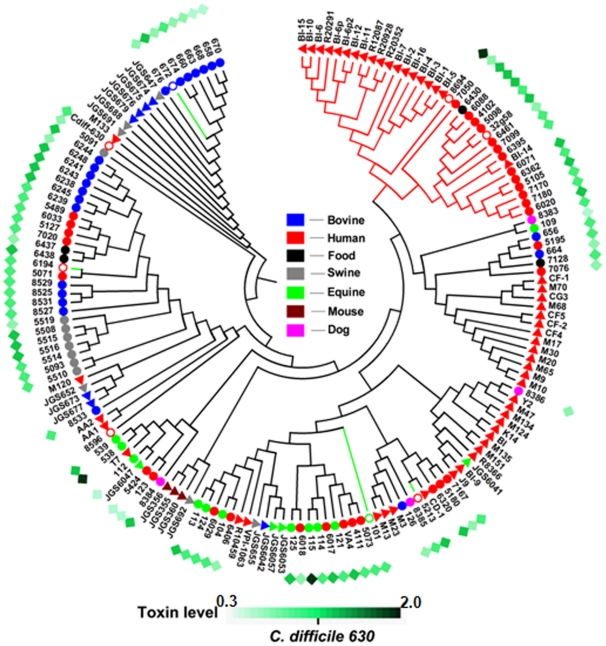
Support tree constructed from CGH data of a total of 167 *C. difficile* isolates. Solid circles and triangles represent strains from our collection and Stabler et al. respectively. Open circles represent sequenced strains. Numbers next to these symbols are strain abbreviation from Figure S1. Color legends for host origin are given at the center of the figure. Hypervirulent clade is marked as a red branch. Strains sequenced in this study are marked as green branch. The diamonds in the outermost ring represent the total toxin estimates of strains from our collection. The toxin intensity scale normalized to *C. difficile* 630 is given at the bottom of the figure.

For transcontinental strain comparison, using the GACK transformed data; we constructed an hierarchical clustering (HCL) support tree (Figure 2). Evidently, each cluster contained *C. difficile* isolates from different host origins, except one (marked as a red branch in Figure 2) that was entirely composed of human isolates. Interestingly, all strains in this cluster belonged to the hypervirulent (HY) clade in Stabler *et al.* [11] and the Group II cluster in Janvilisri *et al.* [12] studies. Except for one strain (BI-14) from Stabler's dataset [11], the strains from both of these previous reports did not mix in this cluster and formed two close sub-clusters under a single branch. However, we considered strains within this major cluster as HY strains because of (i) the similarity between the strains from the Group II in Janvilisri *et al.* study [12] and the HY clade in Stabler *et al*. study [11]; (ii) the association between the outbreaks and the 027/NAP1 phenotype of certain strains in this cluster (#5098, 6088, 32g58, 4102 and 6071); (iii) the clustering of the HY outlier BI-14 strain in Stabler's study [11] with the Group II strains from Janvilisri *et al.* study [12]; and (iv) the clustering of the human strain 8694 (originally designated as CIP 107932) that was isolated in 1984 from a patient with pseudomembranous colitis from Reims, France, with the HY clade strains.

To analyze the relationship between total toxin production and clustering pattern, we measured the total toxin production in the strains from our collection using ELISA. The results showed that there was no correlation between clustering pattern and total toxin production (Figure 2). The highest amount of toxin was produced by the strain #6432. This strain belonged to toxinotype XIV/XV and had an 18 bp deletion in *tcdC* gene [12]. The strain #8694 (CIP 107932) was the second highest toxin-producing strain. Consistently, this strain has been shown to produce more toxin than several 027/NAP1 strains [^19^]. It has also been shown that the 8694 (CIP 107932) strain exhibits a lower sporulation rate compared to other strains [19]. Such strains with a high level of toxin production but a low level of sporulation usually have a poor transmission rate. This may explain the reason why this strain is not reported in recent outbreaks. The third highest toxin production level was found in an equine isolate (#115). This strain however was not included in the HY cluster. Our results showed that certain non-027/NAP1 strains exhibited comparable or higher toxin levels than 027/NAP1 strains, pointing towards the possibility of additional factors responsible for the HY phenotype. Although hypervirulence in *C. difficile* has often been linked to elevated levels of toxin production [9], involvement of other factors such as an increase in sporulation have also been suggested [19]. Consistent with our results, a study of 164 *C. difficile* strains with different PCR ribotypes isolated from patients with different severities of CDAD revealed that there was no correlation between the levels of toxin measured *in vitro* and fecal samples for the corresponding *C. difficile* isolates or between the PCR ribotypes and disease severity [20].

The isolates for which the CGH was conducted exclusively in this study were found to be clustering in different parts of the HCL support tree. The prevalence of *C. difficile* isolated from both farm and companion animals is increasing recently [^21^]. *C. difficile* transmission between animals and human is often suspected as the reason for widespread CDAD incidences [22]. Hence this clustering pattern particularly for the dog isolates is not surprising. Interestingly, one of the food isolates (#6430) was grouped in the HY cluster, suggesting the possibility of food-borne transmission of HY *C. difficile*. The other food isolates were scattered in the HCL tree.

### Genome sequencing of human, bovine and equine isolates

**Figure 3:**
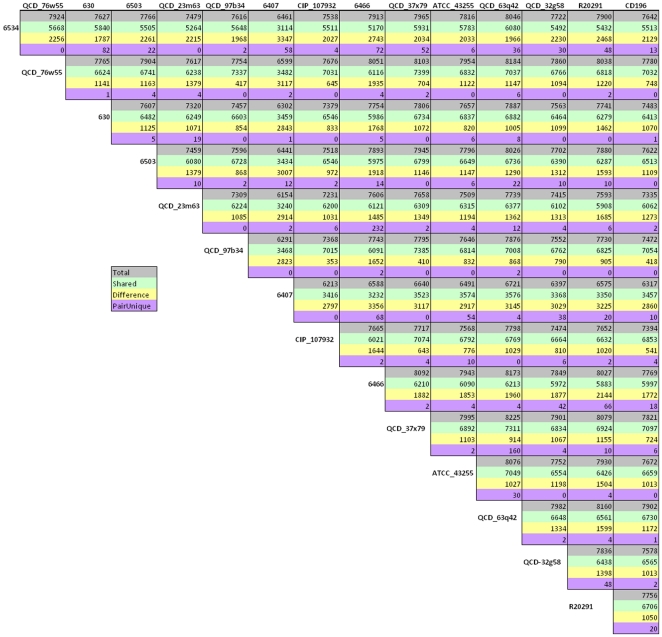
Pairwise genome sequence comparisons. Each of the genome is compared against 14 other genomes. Color-coding designates the following four comparisons between pairs of genomes. Grey; the total number of genes in each pair, green; the number of shared genes in each pair, yellow; the number of genes that are different between the pair, and purple; the number of conserved genes between the pair but absent in all other genomes.

Based on CGH comparison with the reference genome *C. difficile* 630, we selected four isolates with high levels of CDS divergence for sequencing. These included two human (#6466 and 6503), one equine (#6407) and one bovine (#6534) isolates. The sequencing read parameters for the above genomes is given in Table 1. Comparison between these sequenced genomes with other *C. difficile* genomes available in NCBI database showed that the median number of genes for all strains to be 4,047. The number of unique genes (i.e. genes that have no homology in any other genomes compared here) in four strains sequenced in this study was much higher than other sequenced genomes (Table 2). Previous CGH analysis of these four strains showed that ∼700 CDS in these strains were divergent [12]. Hence the discovery of higher number of unique genes in these genomes supports the detection of large number of divergent CDS in CGH comparisons. Since the median number of CDS across the strains is close to the number of CDS in the reference strain, it can be assumed that despite massive variation, the genome size is stable. OrthoMCL program was used to identify orthologues that were shared across all genomes (core genes), orthologues shared between two or more genomes (shared genes) and genes unique to only one genome (unique genes). There were a total of 7,846 genes for all 15 strains. Of these, 1,026 were present across all genomes, 3,864 were shared and 2,956 were singletons. The pairwise comparisons between all 15 genomes are summarized Figure 3. A fasta file containing all the CDS from all strains compared in this study is available in Dataset S1 and S2.

**Table 1:** Summary of sequencing parameters for *C. difficile* genomes sequenced in this study.

Strain	Total number of reads	Total number of bases	Average read length (bp)
6503	401956	90156026	224.3
6466	234489	50759251	216.5
6407	102112	19265037	188.7
6534	182210	38828208	213.1

**Table 2:** Summary of *C. difficile* genomes compared in this study.

Strain	Host origin	Genome size (bp)	Genes	Genbank Accession
630	Human	4290252	3971	AM180355
QCD-97b34	Human	4061547	3748	ABHF00000000
ATCC 43255	Human	3919385	3959	ABKJ00000000
CIP 107932	Human	4056252	3686	ABKK00000000
QCD-23m63	Human	4198222	3611	ABKL00000000
QCD-63q42	Human	4442974	4243	ABHD00000000
QCD-76w55	Human	4395390	4094	ABHE00000000
QCD-37x79	Human	4331780	4082	ABHG00000000
QCD-32g58	Human	3919067	4071	AAML00000000
R20291	Human	4073348	3567	NC_013316
CD196	Human	4006976	3595	NC_013315
6466	Human	3914179	4601	ADDE00000000
6407	Equine	3548389	7328	ADEH00000000
6503	Human	4211360	4024	ADEI00000000
6534	Bovine	4411772	5609	ADEJ00000000

### Prediction of core and pan genome size

**Figure 4:**
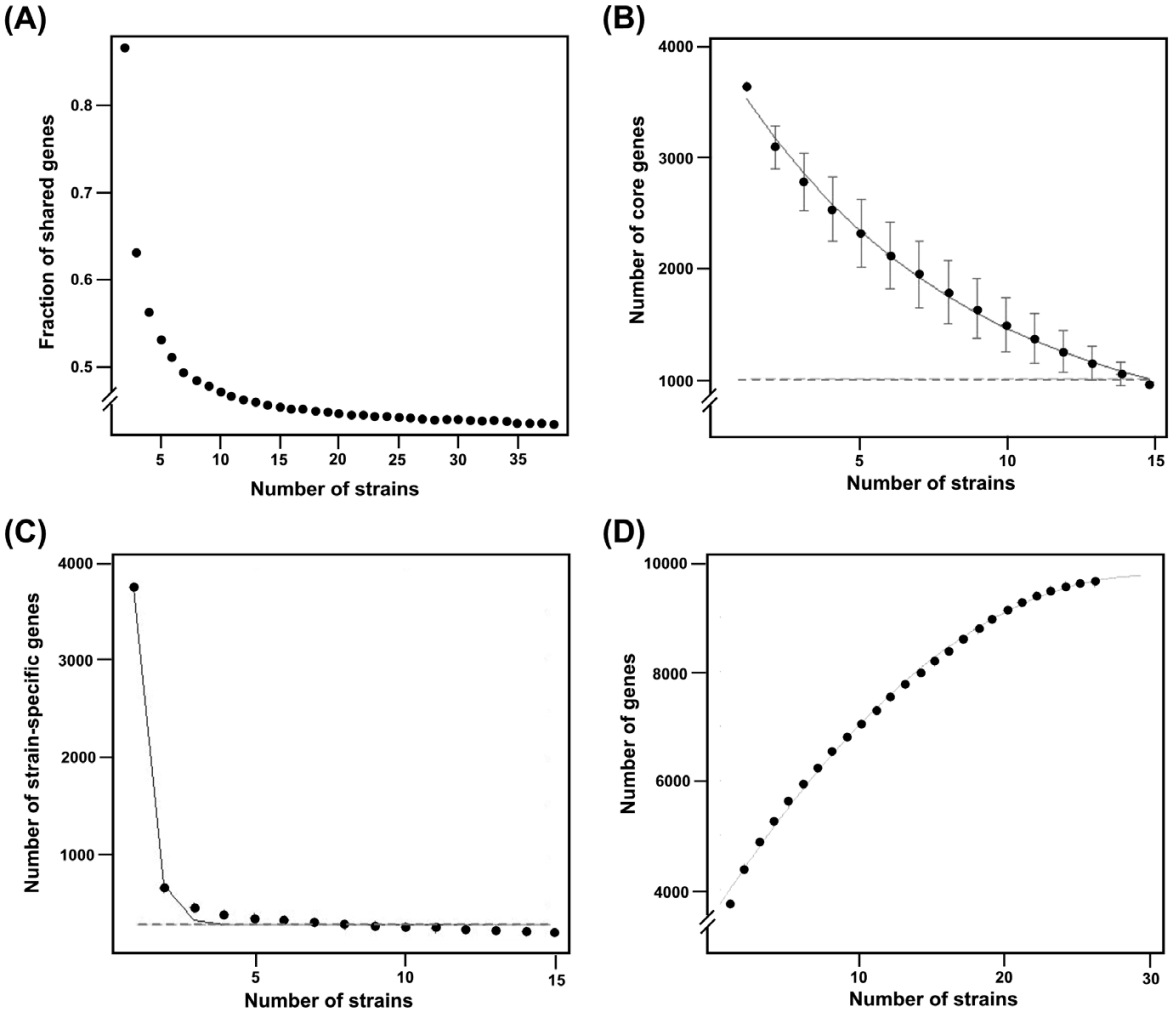
Estimation of core and pan-genome size. The GACK transformed microarray data (Table S2), was used to fit exponential regression function [27] (A) For calculation of core genome size using sequence data, OrthoMCL output after comparing 15 genomes was parsed using custom PERL script and genome sequences were added in random order with 10,000 permutations (B). A plot of the number of new genes per sequenced strain (strain-specific genes) as a function of the number of strains (n) is shown in 4(C). Pan-genome estimate using a cubic function fit is shown in 4(D).

The core and pan genome size was estimated using both microarray and genome sequence data. The accuracy of these estimates depends on the nature of the strains sampled and the sample size, where a larger dataset containing isolates from various hosts from different geographical locations would yield a better prediction. The GACK transformed microarray dataset (Table S2), was used to fit exponential regression function [23]. As expected, the number of core genes reduced dramatically with initial addition of strains in the random sampling, but stabilized with progressive sampling and reached a plateau. The results indicated a core genome size of about 947 genes (23.7% of the total CDS) (Figure 4A).

In order to estimate the core and pan genome size using genome sequence data, we followed a method similar to the one described by Tettelin *et al.* [23]. We sequentially added strains in random order and parsed the OrthoMCL output to calculate (a) the number of genes that have homologous genes in all strains and (b) the number of genes that are “new” to each strain, i.e. those that have no homology in any of the strains. We repeated this for 10,000 random permutations of strain order. As pointed out in a recent review [24], the choice of cut-off points to define the core, unique and shared genes and also the annotation parameters can greatly influence these projections. Hence we used low, medium and high BLAST-P E-values to make the comparisons in OrthoMCL analysis. However, all three parameters returned similar gene estimates. To avoid the effect of differences in the genome annotation, we also re-annotated previously sequenced genomes using the JCVI annotation pipeline. The genes with homology in all strains constitute the “core genome” of *C. difficile*, and a plot of the number of core genes as a function of strain number (n) is shown in Figure 4B. It was observed that the core genome consists of 1,033 genes at n = 15. The difference in the estimated core genome size could be due to the difference in the number of CDS in microarray and sequence data. However, the number of core genes in both predictions is higher when compared to the previous reports [11], [12]. A plot of the number of new genes per sequenced strain (strain-specific genes) as a function of the number of strains (n) is shown in Figure 4C. We found that the number of strain-specific genes was ∼286 (n = 15) with a decreasing trend if the n is higher. Using a cubic function fit in the extrapolation, we found that the number of new genes tended toward zero at n = 26. This indicated that roughly 26 strains were required to capture the entire pan-genome of *C difficile.* We also found that the size of pan genome increased sharply with initial addition of genomes, but seemed to reach a plateau at ∼9,640 genes (Figure 4D).

Although the new core genome projection is higher than the previous estimates [11], [12], it is still much lower than the core genome estimates of other bacterial species. For example, when Tettelin *et al* first introduced the concept of core and pan genome, they estimated the core genome in *Streptococcus agalactiae* to be 80% of the genome [23]. In *S. pneumoniae*, this is estimated as low as 46.5% of the genome [25]. The naturally competent *H. pylori* is projected to have a core genome of 72.5% of the total genome [13] and in *Campylobacter jejuni* it is estimated to be 59.2% [26]. The pan genome estimate of *C. difficile* (9,640 genes) derived from sequence data extrapolation, showed that many more strains are needed to be sequenced to reveal the complete species genome pool. Since the non-epidemic animal and human isolates contained more unique genes than other epidemic strains, we speculate that the “accessory gene pool” available in these non-epidemic strains is being exchanged between strains, thereby enabling adaptive responses to new niches.

### Prediction and curation of pathways in *C. difficile*

In the light of the large-scale genome variation, we analyzed the impact of this variation in the cellular pathways and biochemical networks. Since the perturbation in basic cellular machinery can make cell unviable, reconstruction of cellular pathways can complement sequence-based genome analysis. When such reconstructions are combined with experimental data including transcriptional profiling and proteome sequencing, it cannot only provide a quality control of the assumptions made from the sequence data but also can identify missing enzymes and potential targets for combination therapy [27], [28]. Pathologic program [29] in Pathway tools package [30] was used to construct the cellular pathways of *C. difficile* 630 from its genome sequence. Metacyc was used as the reference database and pathway hole filler in Pathologic was used to infer missing links in pathways [31]. The pathologic program in Pathway tools package is reported to have accuracy comparable to that of an expert biochemist, but exceeds the expert analysis in its comprehensiveness [32]. This automated reconstruction returned a total of 866 enzymes, 1,038 reactions, 828 compounds and 191 pathways. To ascertain our predictions, we then used microarray expression and proteome sequencing data onto the predicted pathways. First, we used the microarray dataset derived from Emerson *et al.* study [33] in which the transcriptional profile of *C. difficile* was analyzed after exposure to antibiotics, pH shift, temperature shift and aerobic shock. When a combined list of significantly differentially expressed (DE) genes following the exposure to antibiotics including amoxicillin, clindamycin and metrinadazole was overlaid onto cellular pathways, it was found that most DE genes were involved in the reactions of cell structure biosynthesis pathways (peptodoglycan biosysnthesis I, II & III, teichoic acid biosynthesis and UDP-N-acetyle-D-glucosamine biosynthesis I). The fact that the antibiotics such as amoxicillin disrupt the cell wall biosynthesis supports our strategy of using high-throughput data for pathway curation. Using the combination of all conditions in the Emerson et al. dataset, a total of 168 pathways were mapped (Table 3). Next, we were able to map a total of 62 pathways using the dataset from a study by our group in which *C. difficile 630* was used to infect Caco-2 cells up to 120 min post-infection [^34^]. Some of these were overlapping with pathways mapped from previous dataset but some were unique to this dataset. Finally, we used data from *C. difficile* membrane protein sequencing [35] and spore proteome sequences [36] to ascertain the predicted pathways. Using the spore protein data, we were able to map 81 pathways whereas membrane protein sequences were mapped onto 32 pathways. All the pathways mapped using membrane protein data were already mapped by spore protein data. When all pathway mappings were converged, we were able to ascertain the presence of 398 enzymes, 584 reactions, 598 compounds and 152 pathways (Table 3). Since several pathways were mapped to more than one dataset, these pathways could be ascertained with high confidence. A summary of the mapped pathways from all datasets is given in Figure S1, and the list of the genes in all datasets used for this analysis is given in Table S4. Similar approaches have been employed in the pathway reconstruction of *H. pylori*, *Vibrio cholerae* and *Leishmania major* [32], [37], [38]. Since we integrated multiple independent datasets into the pathway verification, our results can be viewed with high confidence.

**Table 3:** Summary of pathway prediction and evidence support.

Condition	Pathways	Enzymes	Reactions	Compounds
Total predicted	191	866	1038	828
pH	18	80	119	189
Temperature	62	252	307	368
Antibiotic	88	312	425	471
Caco-2 cell infection	62	265	299	344
Spore protein	81	289	378	423
Membrane proteins	32	121	133	167
Combination of all evidences	152	398	584	598

### Analysis of pathway variation reveals adaptations towards increased virulence

**Figure 5:**
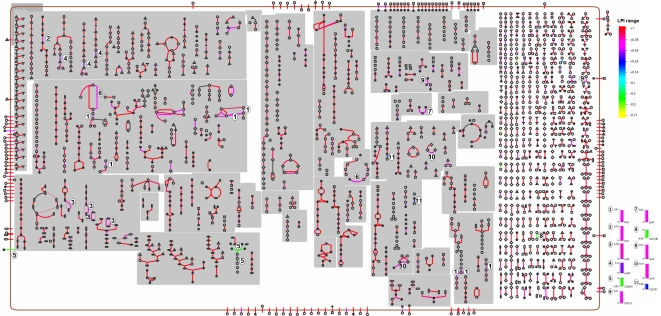
Analysis of pathway variation. LPI values from combined microarray CGH data were overlaid on *C. difficile* 630 pathways. The color scale on the right side of the figure indicates LPI range. Red indicates no variation and yellow indicates maximum variation. Key variable reactions are indicated by numbers on the pathways and their LPI values are given as bar graphs on the right bottom of the figure.

To further characterize the pathways in *C. difficile*, we also analyzed the variation in these pathways by overlaying LPI index for each gene. The results revealed that despite the massive genome variation, almost all cellular pathways in *C. difficile* were conserved. Variation in certain pathways was due to the variability of less than a dozen of enzymes that were part of multiple pathways (Figure 5). The most notable variation was in the biosynthesis pathways of cofactors, prosthetic groups, electron carriers as well as nucleotides and nucleosides. Examples include (i) *folD* (LPI  = 0.6) encoding for the bifunctional protein, a key component of tetrahydrofolate biosynthesis pathway; (ii) *malY* (LPI  = 0.5) encoding a bifunctional protein in the methionine biosynthesis pathway which is coupled with tetrahydrofolate synthesis; (iii) *nrdE* (LPI  = 0.78) encoding a component of ribonucleoside reductase alpha chain; (iv) *CD0244* (LPI  = −0.45) encoding a glycerophosphotransferase in teichoic acid biosynthesis pathway and (v) *erm1b* (LPI  = −0.45) encoding a protein involved in antibiotic resistance. Variation in other pathway components with disposable functions was also found. Interestingly, when the product of *CD0244* was searched against other NCBI bacterial genomes using BLAST-P, the best bidirectional hit was against the gene *HMPREF0542_1838* (hypothetical protein) from *Lactobacillus ruminis E194e* ATCC 25644, indicating that this gene might have been acquired by horizontal transfer. It is surprising to find the variation in tetrahydrofolate biosynthesis indicated by the low LPI values of *folD* and *malY* as this pathway is conserved in all bacteria [39]. Tetrahydrofolate serves as a donor of one-carbon units in a number of biosynthetic processes, including the formation of methionine, purines and thymine. Furthermore, tetrahydrofolate can also act as an acceptor of one-carbon units in degradative reactions [39]. Although all organisms require folate, methods for obtaining them differ between prokaryotes and higher organisms. While mammals possess an active transport system, utilizing membrane-associated folate transport proteins [40], folates must be synthesized *de novo* through the folate biosynthetic pathway in plants and most microorganisms. Hence, folate biosynthesis pathway is usually a target of many antibiotics including sulfonamide and trimethoprims. To exclude the possibility that the low LPI values of these could have resulted from the loss of signal in microarray hybridization due to sequence variability in the gene, we examined the OthoMCL output of all sequenced genomes in this analysis. We found the presence of *folD* in all strains with variation in the nucleotide sequence in some of these genomes.

Genes like *dnaN*, *dnaH*, *gyrB* and *recA* that are associated with DNA replication, recombination and repair pathways were also found to have relatively low LPIs. Fluoroquinolone (FQ) antibiotics such as ciprofloxacin interfere with bacterial growth by causing DNA damage [41], [42]. Surveys now show an alarming pattern of resistance to the majority of FQs currently used in hospitals and outpatient settings with many strains having mutations in *gyrA* [43]. It has been also suggested that ciprofloxacin may induce repair pathways that involve RecA-ssDNA filament formation; the drug itself may act to induce the mutations that confer resistance [44]. Hence the sequence variation in genes like *gyrB* and *recA* could be the result of FQ therapy to treat *C. difficile* infection.

The conservation in the cellular pathways was also confirmed by comparing the presence of these genes in all sequenced genomes. The comparisons showed the presence of the genes with high LPI values in all sequenced genomes, indicating that the pathway preservation indicated by microarray data also reflected in the sequence level data. Since some sequence variation was found for genes like *gyrB* and *recA* in sequenced genomes, the distribution of all the genes constituting homologous recombination, mismatch repair, nucleotide excision repair, base excision repair and DNA replication machinery was examined across all sequenced genomes. The results showed that all of these pathways and most of the genes constituting these pathways were conserved across the genomes (Table S5). Although the link between antibiotic exposure and bacterial sequence variation in genes such as *gyrB* and *recA* has been established, evidence for the role of sequence variation in genes such as *dnaN* and *dnaH* antibiotic treatment is scanty. It has been shown that in *E. coli*, ciprofloxacin induced damage is countered by induction of nucleotide excision repair (NER), homologous recombination (HR) and SOS response [42]. Hence it is possible that the variation in *dnaN* in *C. difficile* could be the result of a recombination-mediated mechanism to counteract the action of antibiotic like ciprofloxacin. However, further investigations are required to clarify this probability.

## Materials and Methods

### Genomic DNA extraction, microarray hybridization, data analysis and support tree construction

Genomic DNA extraction, DNA labeling and microarray CGH of food, dog and bovine isolates against *C. difficile 630* DNA was carried out using a custom array (GEO platform ID GPL6118) as described previously [12]. CGH data from this study were submitted to GEO (accession number GSE19343). CGH data of 73 *C. difficile* isolates hybridized against *C. difficile 630* DNA was downloaded from GEO Series GSE9693 [12]. Normalized CGH data for 75 *C. difficile* isolates from Stabler *et al* [11] was downloaded from ArrayExpress (accession number E-BUGS-41). The complete detail of all strains used in this analysis is given in Table S1. To place the reference strain *C. difficile 630* in comparison tree, self-CGH was performed. After normalization, the genes in the whole dataset (167 strains in total) were classified as present, absent or divergent using 100% EPP cutoff using GACK algorithm [45]. For transcontinental strain comparison, a support tree was constructed using HCL support tree algorithm in Mev [46]. Trees were re-sampled by jackknifing with 1,000 iterations with Pearson correlation as the distance matric and complete linkage rule. The resulting tree was exported in Newic format and modified in MEGA 4.0 [47].

### Calculation of locus plasticity index

GACK algorithm does not give information on the degree of divergence of individual genes in the divergent category [45]. To numerically represent the level of divergence in each CDS, we devised a new index named Locus Plasticity Index (LPI) from GACK transformed data; calculated as
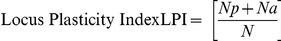



Where, *Np* =  number of present loci, *Na* =  number of absent loci and *N* =  number of present, absent and divergent loci.

### Measurement of total toxin production

Strains were stocked in −80°C and were inoculated into modified BHI broth. BHI broth was prepared from Difco™ Brain Heart Infusion. For reducing the medium, 20 ml/L of Oxyrase® for broth was added and the medium was incubated at 37°C in an anaerobic chamber overnight. To ensure equilibration of all strains in the growth medium, all strains were sub-cultured three times. 1 ml was withdrawn at 48 h post-inoculation from the third culture and was then passed through 0.22-µm membrane. 200 µl of the filtrate was then used for total toxin quantification using Premier Toxin A&B ELISA kit following the manufacture's protocol (Meridian Bioscience Inc. Ohio). To normalize any cell density differences in between strains growth and toxin levels, each strains OD_600_ was measured and ELISA intensity values were divided by corresponding OD_600_ values. Two biological replicates were performed and the means of ELISA intensity were taken as the final toxin level. For comparative analysis, ELISA intensities of all strains were divided by the reference strain *C. difficile* 630 ELISA intensity.

### Pathway reconstruction, curation and analysis of pathway variation

For reconstruction of *C. difficile* pathways, the complete genome sequence of the reference strain *C. difficile* 630 was downloaded from Genbank (accession number AM180355). Using Pathologic tool from Pathway tools software [30], [48], the complete cellular pathways were predicted. Pathway hole filler program [29] was used to fill the missing links or holes in the predicted pathways. This program utilizes the BLAST searches of a set of sequences encoding the required activity in other genomes to identify candidate proteins in the genome of interest, in addition to genomic context (such as the candidate enzyme being in the same operon as another gene in the pathway) to determine the probability that a candidate enzyme has the required function [29]. Automatically predicted pathways were then curated manually using microarray expression and proteome data. A list of genes in *C. difficile* 630 whose expression changed significantly in different transcriptional conditions was obtained from Emerson *et al* study [33]. A second set of genes that were detected to be active at protein level was derived from *C. difficile* 630 membrane proteome analysis conducted by Wright *et al* [35]. Finally genes that were coding for *C. difficile* spore were obtained from spore proteome sequencing conducted by Lawley *et al* [36]. A pathway was deemed present when any gene from the above datasets mapped to that pathway. Cellular Omics viewer program [48] in Pathway tools was used for overlaying the LPI values for all *C. difficile* CDS on to the curated pathways.

### Genome sequencing, assembly, annotation and comparative analysis

Based on the CGH results, we selected four strains with large number of variant CDS (strains 6407, 6466, 6503 and 6534) for genome sequencing. Genomic DNA of these strains was sequenced by 454 Life Sciences GS-20 sequencer using standard protocols [49]. Using non-paired end sequencing chemistry, strains were sequenced up to a depth of 15x and assembled *de novo* by the 454 Newbler assembler. Contigs were ordered and oriented based on their alignment to the reference genome *C. difficile* 630 using NUCMER. Contigs thus ordered and those matching to reference sequence were joined together into a pseudochromosome, and non-matching contigs were added at the end in random order. A linker sequence (NNNNN CAT TCC ATT CAT TAA TTA ATT AAT GAA TGA ATG NNNNN) that provided start and stop codons in all six reading frames permitting the identification of genes that extend past the ends was used to join the contigs. The pseudochromosome for each strain was submitted to the J. Craig Venter Institute (JCVI) annotation service, where it was run through JCVI's prokaryotic annotation pipeline. Included in the pipeline is gene finding with Glimmer, Blast-extend-repraze (BER) searches, HMM searches, TMHMM searches, SignalP predictions, and automatic annotations from AutoAnnotate. All of this information is stored in a MySQL database and associated files which were downloaded to our site. The other 11 genomes included in this analysis were downloaded from NCBI, thus constituting a total of 15 strains in the comparative analysis (Table 2). Using coding sequences from each strain, orthologs were determined and clustered using OrthoMCL[50]. OrthoMCL was run with a BLAST E-value cut-off of 1e-5, and an inflation parameter of 1.5. Custom PERL scripts were used to parse the OrthoMCL output for cluster information and pair wise strain comparisons. Core and pan-genome size was estimated following the methods described by Tettelin *et al* [23]. Core genome size was calculated using both microarray data and OrthoMCL output and pan-genome size was calculated using OrthoMCL output alone.

## Supporting Information

Table S1 **A list of strains used in CGH comparisons.**
(XLS)

Table S2 **A list containing GACK transformed microarray data for all strains.**
(XLS)

Table S3 **A list containing LPI values for all genes in** ***C. difficile.***
(XLS)

Table S4 **A list of DE genes from all microarray and proteome sequencing that were used for annotating** ***C. difficile*** **pathways.**
(XLS)

Table S5 **A list containing LPI of genes involved in recombination, mismatch repair, nucleotide excision repair, base excision repair and DNA replication machinery of** ***C. difficile.***
(XLS)

Dataset S1 **A text file containing all gene sequences in FASTA format from all 15 strains used for genome comparisons.**
(RAR)

Dataset S2 **A text file containing all gene sequences in FASTA format from all 15 strains used for genome comparisons (continuation of Dataset S1).**
(RAR)

Figure S1 **A pdf file containing a figure showing overview of different** ***C. difficile*** **pathways annotated using all data sources.**
(PDF)
