# Supplementary material for: Analysis of Ultra Low Genome Conservation in Clostridium difficile
Source: PLoS One. 2010 Dec 8;5(12):e15147. doi: 10.1371/journal.pone.0015147 (PMC2999544; doi:10.1371/journal.pone.0015147)

Additional data file 5.

Figure below shows the overview of high-throughput data evidence for *C. difficile* pathways. Significantly differentially expressed genes from microarray experiments and proteome sequencing were overlaid onto cellular pathways of *C. difficile* 630. Line colors denote different comparisons and data sources as per the following scheme: Blue = reactions and transporters predicted by pathologic, but were not mapped to any of the experimental data. Color legends are as follows; Red = pathways and transport functions shared with other pathogenic clostridia. Orange = pathways and reactions corresponding to antibiotic stress, Green = pathways and reactions corresponding to pH shift, Pink = pathways and reactions changing during Caco-2 cell infection, Dark brown = pathways and reactions corresponding to aerobic shift, Light green = pathways and reactions changing during heat shock, Yellow = pathways and reactions detected from sequencing *C. difficile* 630 membrane proteins, Greenish brown = pathways and reactions detected from sequencing *C. difficile* 630 spore proteins.

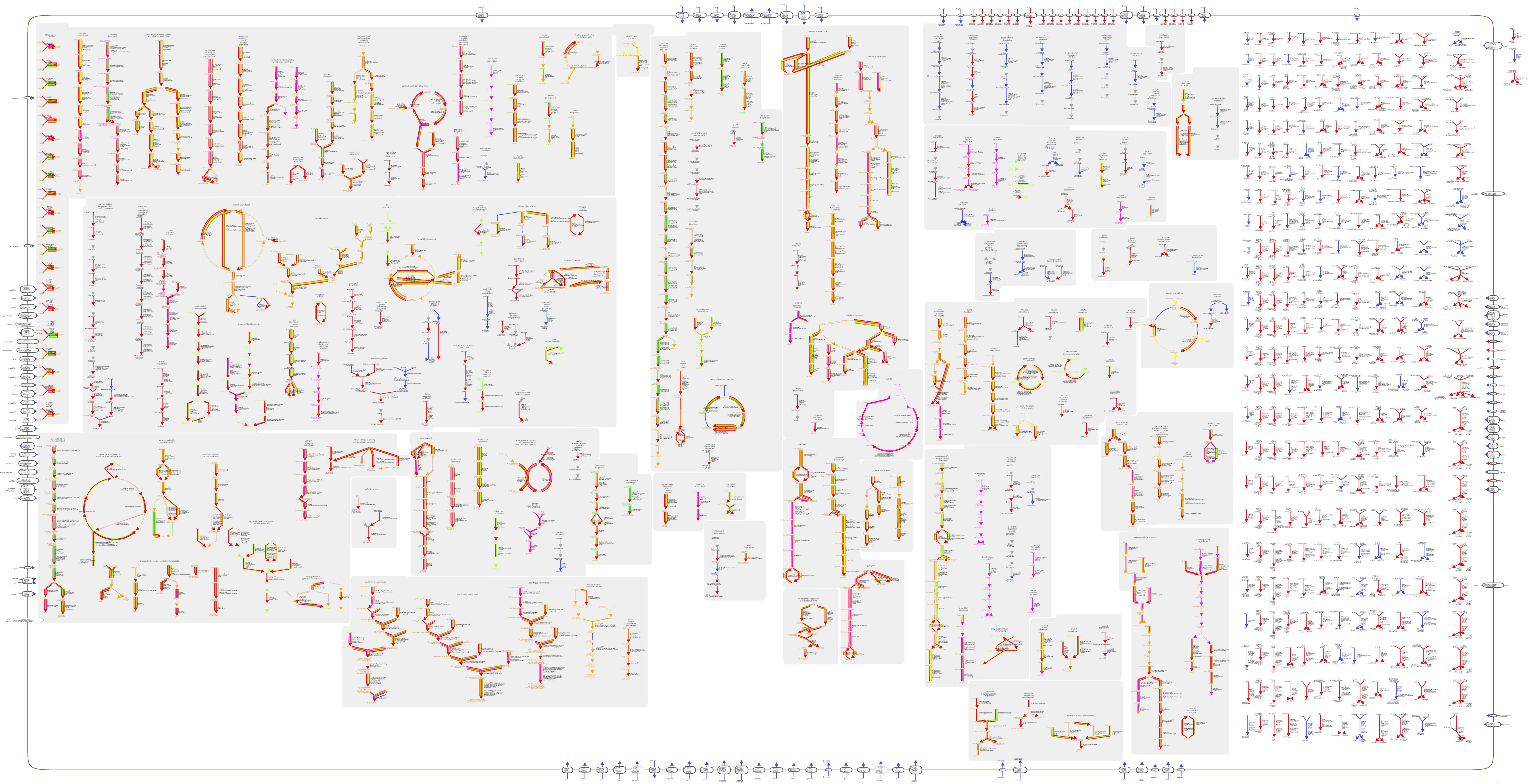

Supplement: Figure S1 — A pdf file containing a figure showing overview of different C. difficile pathways annotated using all data sources. (PDF) [file pone.0015147.s008.pdf]
